# Unequal availability of workplace policy for prevention of coronavirus disease 2019 across occupations and its relationship with personal protection behaviours: a cross-sectional survey

**DOI:** 10.1186/s12939-021-01527-x

**Published:** 2021-09-07

**Authors:** Kailu Wang, Eliza Lai Yi Wong, Kin Fai Ho, Annie Wai Ling Cheung, Emily Ying Yang Chan, Samuel Yeung Shan Wong, Eng Kiong Yeoh

**Affiliations:** 1 Centre for Health Systems and Policy Research, JC School of Public Health and Primary Care, Faculty of Medicine, The Chinese University of Hong Kong, Hong Kong, China; 2 JC School of Public Health and Primary Care, Faculty of Medicine, The Chinese University of Hong Kong, Hong Kong, China

**Keywords:** COVID-19, Prevention, Workplace policy, Personal protection behaviour, Occupation

## Abstract

**Background:**

The evolving pandemic of coronavirus disease 2019 (COVID-19) has become a severe threat to public health, and the workplace presents high risks in terms of spreading the disease. Few studies have focused on the relationship between workplace policy and individual behaviours. This study aimed to identify inequalities of workplace policy across occupation groups, examine the relationship of workplace guidelines and measures with employees’ behaviours regarding COVID-19 prevention.

**Methods:**

A cross-sectional online survey using a structured questionnaire was conducted to gather employees’ access to workplace guidelines and measures as well as their personal protection behaviours. Statistical associations between these two factors in different occupations were examined using multiple ordinal logistic regressions.

**Results:**

A total of 1048 valid responses across five occupational groups were analysed. Manual labourers reported lower availability of workplace guidelines and measures (76.9% vs. 89.9% for all, *P* = 0.003). Employees with available workplace guidelines and measures had higher compliance of hand hygiene, wearing masks, and social distancing, and this association was more significant among managers/administrators and manual labourers.

**Conclusions:**

Protection of the quantity and quality of employment is important. Awareness about the disease and its prevention among employers and administrators should be promoted, and resources should be allocated to publish guidelines and implement measures in the workplace during the pandemic. Both work-from-home arrangement and other policies and responses for those who cannot work from home including guidelines encouraging the health behaviours, information transparency, and provision of infection control materials by employers should be established to reduce inequality. Manual labourers may require specific attention regarding accessibility of relevant information and availability of medical benefits and compensation for income loss due to the sickness, given their poorer experience of workplace policy and the nature of their work. Further studies are needed to test the effectiveness of specific workplace policies on COVID-19 prevention.

**Supplementary Information:**

The online version contains supplementary material available at 10.1186/s12939-021-01527-x.

## Background

Coronavirus disease 2019 (COVID-19) is an illness caused by a novel pathogen that has been named “severe acute respiratory syndrome coronavirus 2” (SARS-CoV-2) [1]. As of early April 2020, over 1.4 million COVID-19 cases and 80,000 deaths have been reported in over 100 countries and regions worldwide after the first cluster of cases was reported in Wuhan, China [2]. The World Health Organization (WHO) declared the situation as pandemic on 11 March 2020 [3]. Patients with COVID-19 can present a wide spectrum of symptoms and severity, ranging from no fever or abnormality on lung radiology results to multiple complications [1]. Human-to-human transmission has been confirmed by numerous studies [4, 5]. A cluster of cases in hospital and family settings [1] suggests that the disease can be easily spread between people who have close contact with each other, including work-related settings. In early March 2020, the media reported an outbreak of COVID-19 in a company meeting in Massachusetts, US, with 70 out of the first 92 COVID-19 cases in Massachusetts being linked to employees of this company by 10 March 2020 [6]. This case demonstrated how severe the consequences of an outbreak in the workplace can be; therefore, more attention should be paid to the guidelines and measures provided at the workplace to prevent the disease.

To respond to the evolving pandemic, multiple types of interventions and policies have been implemented by governments in various countries and regions, including school and workplace closures, cancelling public events and public transport, public information campaigns, and restrictions and control of internal movement and international travel, as summarized by a database established by the University of Oxford [7]. Among these policies, the closure of certain workplaces, compulsory and recommended work-from-home policies, and flexible work hours were announced by several governments for the purpose of social distancing. Multiple health organizations and authorities also published their own guidelines for employers and employees to prevent COVID-19 spread in the workplace. The WHO suggested a set of guidelines for employers to prepare and respond to COVID-19 in their workplace [8], which included advice for maintaining good personal hand hygiene, maintaining social distancing in the workplace, through suggestions such as holding online meetings instead of face-to-face ones and precautions for work-related travel. Online resources from the WHO and the International Labor Organization have also been synthesized and provided by the European Agency for Safety and Health at Work (EU-OSHA) [9]. Most of them focus on preventing infection in healthcare facilities for health workers and public emergency workers, which is essential for maintaining the capacity and efficiency of the health system. However, the information and resources provided for non-health and ordinary workplaces have been limited, even though some of these occupations involve exposure to crowds, longer working hours, and lower income. It was reported in 2019 that the median monthly salary for craft workers, machine operators, elementary workers, and sales/service workers in Hong Kong (HK) ranged from HK$12,900 to HK$21,900 (around US$1654–2808) while it was HK$28,100 (around US$3603) for managers, professionals, and associate professionals; moreover, the median number of weekly working hours was 48.0 h for manual laborers, while it was 40.6 h for other occupations [10].

In early April, the EU-OSHA published guidance to assist employers providing advice to staff for adequate prevention [11]. The Centre for Disease Control and Prevention (CDC) in the US also developed guidelines for non-health settings, helping individuals to identify risks and isolate sick employees, promoting social distancing and hand and environment hygiene, and encouraging employers to provide health education for employees [12]. Thus, the focus on COVID-19 prevention in non-health workplaces hasincreased. However, a considerable part of the contents of the aforementioned policies, guidelines, and instructions aims to promote prevention in the workplace by altering and improving the personal protection behaviours of employees, such as hand and environment hygiene and social distancing. The macro-level guidelines published by governments and health authorities might not catch the attention of, or be easily accessed by, ordinary employed individuals.

More importantly, the unequal availability of workplace guidelines and measures for COVID-19 prevention across occupations should be emphasized. Previous studies have revealed that individuals living in more deprived areas are more vulnerable to community outbreaks of COVID-19 as well as more easily affected by responses to pandemic [13, 14]. Inequalities in housing condition may also contribute to inequalities in incidences of COVID-19 [15]. However, the inequality across occupations has received less attention in the literature of COVID-19, despite inequalities in mortality in different occupations have been found in the 1918 influenza pandemic and many chronic illnesses [15]. There are only a few studies focusing on this issue among the working population. A US national survey during 2014–2017 indicated that there were racial/ethnic differences in occupations at high risks of COVID-19 severe illness [16]. Another US-based study found that Hispanic or non-white workers in wholesales, manufacturing, transportation, accommodation and food services were facing greater work-associated infection of COVID-19 than those working in professional, finance, public administration, and entertainment occupations [17]. These studies highlighted the disparities of COVID-19 infection risks across occupation groups but neither of them indicated its relationship with their workplace guidelines. Considering all these, it is necessary to investigate the inequality of relevant workplace guidelines and measures across occupations.

COVID-19 prevention in the workplace is also crucial among high density population areas like HK, a city with 7.45 million residents, a labour force of 3.98 million, and 3.87 million employed persons in 2018, including employers, employees, self-employers, and unpaid family workers [18]. The consequences could be severe if precautions were not taken seriously by employers and employees. Only one local study has been published on the degree of stress and views towards workplace supportive policies and protective equipment supply among employees [19]. To our knowledge, evidence on the relationship between the implementation of workplace guidelines and its relationship with protection behaviours for preventing COVID-19 spread at the corporate level is scarce. Thus, this study aimed to examine the relationship between workplace guidelines and measures and employees’ protection behaviours for COVID-19 prevention and whether behavioural responses differ according to the nature of their occupations. We hypothesized that the availability of workplace guidelines and measures and its associations with personal protection behaviours are different across occupations.

## Methods

A cross-sectional self-administered questionnaire survey of employees was conducted using an online platform from 17 to 27 February 2020 in HK. During the survey period, daily COVID-19 confirmed cases were lower than 10 [20] but the cumulative cases had increased to 93 by the end of February in HK.

### Study sample and data collection

This study focused on the employed population of HK, which comprises 3.54 million individuals [18]. We considered nine occupation groups as defined by the government: 1) managers and administrators, 2) professionals, 3) associate professionals, 4) clerical support workers, 5) service and sales workers, 6) craft and related workers, 7) plant and machine operators and assemblers, 8) elementary occupations and 9) farm workers, animal husbandry workers and fishermen, and other unidentifiable occupations. For this study, HK residents aged 18 years or above, employed or self-employed, working either on a full-time or part-time basis, and able to understand Chinese were eligible. An electronic device was also needed by survey participants for accessing the internet and completing the online questionnaire, which was feasible because of wide possession of web-accessible mobile devices among working-age individuals (over 99% for those aged 15–54 and around 95% for those aged 55–64) [21]. Students, housewives, and those who were retired were excluded, as they did not work in a workplace.

The participants were recruited using the convenience sampling method. The questionnaire was distributed on an online platform to the target population through email and multiple social networking platform, as it is considered to be a safer way for both research staff and participants to avoid contact in person while conducting the survey and reduce the risk of COVID-19 infection. The questionnaire was available as a Google Form and self-administered. An information sheet about the study aims, data collection procedure and participant’s right was included at the beginning of the survey, followed by an electronic consent form. The data collected through the questionnaire were stored in an online drive and password-protected. The people who received the link for the online survey were encouraged to forward the survey links to others.

### Instruments and measures

The questionnaire was developed based on the WHO guidelines for COVID-19 prevention in the workplace [8], and the validated instrument in a related study on health behaviour practice during Ebola epidemics [22] was used to inform identification and categorization of relevant personal protection behaviours for analysis, as its instrument synthesized the findings and instruments from multiple relevant studies under a similar context to the early stage of COVID-19 epidemics when our study was done. This study focused on two major aspects of the survey as follows: 1) availability of workplace guidelines and measures for preventing COVID-19; and 2) frequency of personal protection behaviours in the past 7 days. For the protection behaviours, a total of eight items under four categories were assessed: 1) hand hygiene (handwashing before meals, handwashing after using the toilet, use of alcohol-based hand rub when outside), 2) face mask (wearing face mask when outside), 3) household hygiene (putting disinfectant into toilets, putting disinfectant into drain-pipe), and 4) social distancing (avoiding leaving home, avoiding contact with neighbours). Demographic and occupation information were also collected. A four-point Likert scale (1 = “never”, 2 = “when needed”, 3 = “sometimes” and 4 = “always”) was applied to measure the frequency of personal protection behaviours of the respondents. Regarding the availability of workplace guidelines and measures, respondents provided a binary response: “available” or “not available.”

### Statistical analysis

Data processing and analyses were conducted using Stata 14.0. The demographic and occupational characteristics of survey participants were described and reported. A two-way cross tabulation analysis was performed to assess the association of occupation group with frequency of personal protective behaviours and availability of workplace guidelines, respectively, using chi-square or Fisher’s exact test to identify differences in behaviour frequency and availability of guidelines between different occupation groups. Following this, ordinal logistic regressions for different personal protection behaviours were conducted for all survey participants (i.e. one regression model for each of the eight behaviours) and for participants in different occupation groups separately (i.e. 40 regression models, for all combinations of eight behaviours and five occupations), which can be specified as.

1 $$ \ln \frac{\Pr \left({Y}_{m,i}>j|\ X\right)}{1-\Pr \left({Y}_{m,i}>j|\ X\right)}={\alpha}_{m,j}+\sum \limits_{k=1}^h{\beta}_{m,k}{X}_{k,i}+{\upvarepsilon}_{m,i},\kern2.5em j=1,2,\dots, J-1. $$

where the frequency of behaviour m (Y_m_) with J categories of responses of individual i was the dependent variable, and the independent variables X_k_ included availability of workplace guidelines and measures, occupations (in the models for all survey participants only), age, gender, marital status, living arrangement, education attainment, and work status (full- or part-time), in order to determine the associations between workplace policy and personal protection behaviours and examine the differences in these associations according to occupation.

As the study sample had a disproportionate distribution of occupation compared with the entire working population in HK [18], a sensitivity analysis was conducted using the sample with the adjusted occupation distribution to explore whether the findings from the regression analysis were affected by this disproportion. The adjustment was made by giving different weightings to participants with different occupations based on the relative occupation distribution of the working population in HK. The same multiple ordinal logistic regression models applied in original sample (i.e. eq. 1) were conducted again with the weighted sample for the comparison analysis.

## Results

### Demographics

**Fig. 1:**
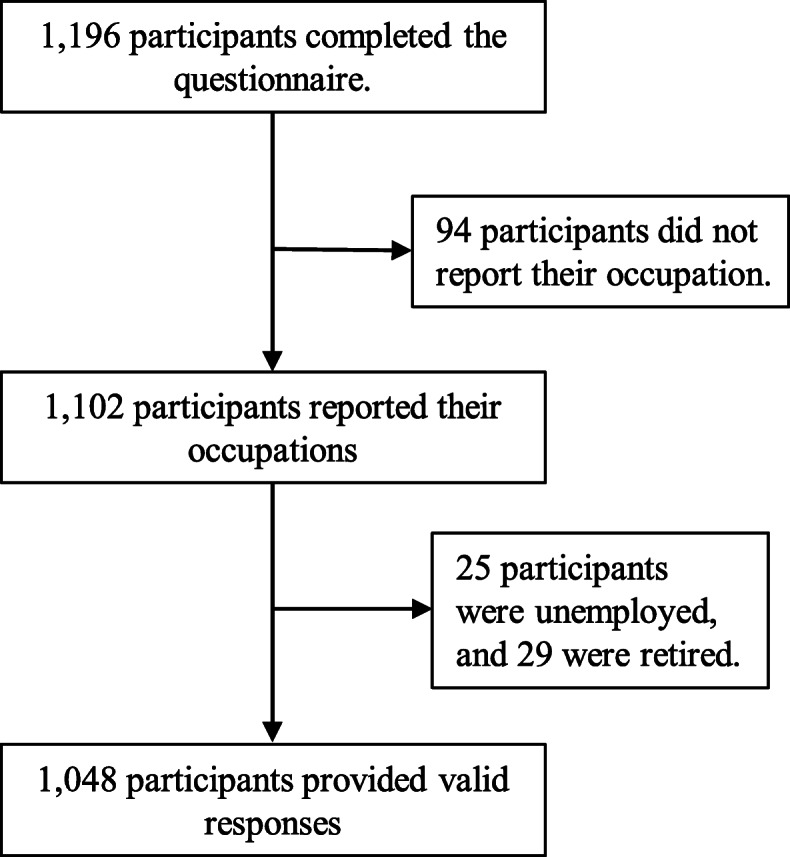
Selection of participants with valid responses

A total of 1196 participants completed the online survey; of these, 148 reported being retired or currently unemployed, or did not provide their occupation. Thus, the responses from 1048 participants were deemed valid for subsequent analysis (Fig. 1 and Table 1). Among the 1048 participants, 68% were female; regarding age, 20.7% were 18–29 years, 28.0% 30–39 years, 32.9% 40–49 years, 15.6% 50–59 years, and 2.8% 60 years and over. Over half of the participants (53%) were married or cohabited, and only 6% were living alone. Around 75% had attained undergraduate education at university or above. The majority of participants (91%) worked on a full-time basis. With regards to occupation (Table 2), 42% were professionals, 24% associated professionals, 17% managers or administrators, and 10% clerical support workers. Our study sample comprised fewer participants from worker groups and more from professional groups according to statistics on the labour force and employed population in HK [18]. For the analysis, the four occupation groups of 1) service/sales/craft workers, 2) plant/machine operators and assemblers, 3) elementary workers, and 4) farm workers, animal husbandry workers and fishermen, and unidentifiable occupations were grouped as “manual labourers,” who comprised around 6% of the study sample (Table 1). Compared with the local working population [18], this study sample did not have a substantial difference in age and gender distribution (working population: 50.5% were female; 18.6% were 18–29 years, 25.0% 30–39 years, 23.6% 40–49 years, 22.7% 50–59 years, and 10.1% 60 years and over), but the occupation distribution was disproportionate. There were more professionals (43.0% vs 7.9%) and less manual labourers (6.3% vs 47.0%) than in the general working population.

**Table 1:** Socio-demographics characteristics and personal protection behaviours of survey participants

	Hand Hygiene			Face Mask	Household Hygiene		Social distancing		
	“Always” hand wash before meals	“Always” hand wash after toileting	“Always” use alcohol hand rub	“Always” wear mask	“Always” put disinfectants into toilets	“Always” put disinfectants in drain pipe	“Always” avoid leaving home	“Always” avoid contact neighbours	Total
	N (%)	N (%)	N (%)	N (%)	N (%)	N (%)	N (%)	N (%)	N (%)
**Age group**									
18–29	150 (17.0)	213 (20.8)	117 (19.8)	209 (20.9)	38 (18.2)	32 (18.5)	100 (18.7)	136 (20.5)	217 (20.7)
30–39	234 (26.6)	291 (28.5)	170 (28.8)	281 (28.1)	46 (22.0)	44 (25.4)	154 (28.7)	183 (27.6)	294 (28.0)
40–49	273 (31.0)	337 (33.0)	206 (34.9)	330 (33.0)	80 (38.3)	62 (35.8)	183 (34.1)	223 (33.7)	345 (32.9)
50–59	126 (14.3)	154 (15.1)	82 (13.9)	154 (15.4)	39 (18.7)	29 (16.8)	82 (15.3)	101 (15.3)	163 (15.6)
60+	25 (2.8)	27 (2.6)	16 (2.7)	26 (2.6)	6 (2.9)	6 (3.5)	17 (3.2)	19 (2.9)	29 (2.8)
*P-value*^*1*^	0.049*	0.014*	*0.227*	*0.471*	*0.147*	*0.353*	*0.684*	*0.279*	
**Gender**									
Female	560 (63.6)	695 (68.0)	441 (74.6)	684 (68.4)	149 (71.3)	124 (71.7)	390 (72.8)	474 (71.6)	712 (67.9)
Male	248 (28.2)	327 (32.0)	150 (25.4)	316 (31.6)	60 (28.7)	49 (28.3)	146 (27.2)	188 (28.4)	336 (32.1)
*P-value*^*1*^	*0.357*	*0.819*	< 0.001**	*0.425*	*0.055*	*0.424*	0.002**	0.005**	
**Marital status**									
Never married	319 (36.3)	437 (42.8)	240 (40.6)	425 (42.5)	83 (39.7)	66 (38.2)	212 (39.6)	275 (41.5)	444 (42.4)
Married	438 (49.8)	518 (50.7)	312 (52.8)	510 (51.0)	106 (50.7)	95 (54.9)	291 (54.3)	347 (52.4)	534 (51.0)
Cohabiting	14 (1.6)	22 (2.2)	12 (2.0)	20 (2.0)	5 (2.4)	4 (2.3)	12 (2.2)	10 (1.5)	23 (2.2)
Divorced/ separated	31 (3.5)	38 (3.7)	23 (3.9)	38 (3.8)	12 (5.7)	5 (2.9)	19 (3.5)	27 (4.1)	39 (3.7)
Windowed	6 (0.7)	7 (0.7)	4 (0.7)	7 (0.7)	3 (1.4)	3 (1.7)	2 (0.4)	3 (0.5)	8 (0.8)
*P-value*^*1*^	0.011*	*0.113*	*0.806*	< 0.001**	*0.518*	*0.592*	0.008**	< 0.001**	
**Living arrangement**									
Alone	49 (5.6)	63 (6.2)	33 (5.6)	60 (6.0)	11 (5.3)	9 (5.2)	32 (6.0)	41 (6.2)	68 (6.5)
With family	733 (83.3)	921 (90.1)	537 (90.9)	905 (90.5)	186 (89.0)	158 (91.3)	481 (89.7)	596 (90.0)	942 (89.9)
With others	26 (3.0)	38 (3.7)	21 (3.6)	35 (3.5)	12 (5.7)	6 (3.5)	23 (4.3)	25 (3.8)	38 (3.6)
*P-value*^*1*^	*0.323*	*0.202*	0.044*	< 0.001**	0.007**	0.037*	*0.788*	*0.121*	
**Education attainment**									
Lower secondary school	4 (0.5)	8 (0.8)	5 (0.8)	9 (0.9)	3 (1.4)	3 (1.7)	4 (0.7)	6 (0.9)	10 (1.0)
Higher secondary school	78 (8.9)	100 (9.8)	54 (9.1)	98 (9.8)	17 (8.1)	15 (8.7)	57 (10.6)	68 (10.3)	103 (9.8)
Preparatory Course	119 (13.5)	144 (14.1)	88 (14.9)	143 (14.3)	28 (13.4)	21 (12.1)	75 (14.0)	87 (13.1)	148 (14.1)
Undergraduate or above	607 (69.0)	770 (75.3)	444 (75.1)	750 (75.0)	161 (77.0)	134 (77.5)	400 (74.6)	501 (75.7)	787 (75.1)
*P-value*^*1*^	*0.280*	*0.084*	*0.944*	*0.563*	0.034*	*0.538*	*0.480*	*0.816*	
**Work status**									
Full-time	735 (83.5)	927 (90.7)	535 (90.5)	907 (90.7)	191 (91.4)	155 (89.6)	477 (89.0)	587 (88.7)	950 (90.7)
Part-time	73 (8.3)	95 (9.3)	56 (9.5)	93 (9.3)	18 (8.6)	18 (10.4)	59 (11.0)	75 (11.3)	98 (9.4)
*P-value*^*1*^	*0.838*	*0.942*	*0.812*	*0.866*	*0.276*	*0.511*	*0.226*	0.017*	
**Workplace policy**									
Available	738 (83.9)	923 (90.3)	536 (90.7)	905 (90.5)	187 (89.5)	154 (89.0)	485 (90.5)	600 (90.6)	942 (89.9)
Not available	70 (8.0)	99 (9.7)	55 (9.3)	95 (9.5)	22 (10.5)	19 (11.0)	51 (9.5)	62 (9.4)	106 (10.1)
*P-value*^*1*^	0.030*	0.027*	*0.740*	0.004**	*0.996*	*0.980*	*0.465*	*0.339*	
**Total**	880 (100.0)	1022 (100.0)	591 (100.0)	1000 (100.0)	209 (100.0)	173 (100.0)	536 (100.0)	662 (100.0)	**1048(100.0)**

*Note*: 1. These *P* value came from Chi-square test or Fisher’s exact test

* *P* < 0.05; ** *P* < 0.01

**Table 2:** Personal protection behaviour and workplace policy among people with different occupations

		Manager and administrator	Professional	Associate professional	Clerical support worker	Manual labourer^**1**^	Total	***P***-value^**2**^
		***N*** = 183 n (Col%)	***N*** = 440 n (Col%)	***N*** = 256 n (Col%)	***N*** = 104 n (Col%)	***N*** = 65 n (Col%)	***N*** = 1048 n (Col%)	
**Hand Hygiene**	**Hand wash before meals**							
	Never	0 (0.0)	2 (0.5)	0 (0.0)	1 (1.0)	0 (0.0)	3 (0.3)	0.736
	When needed	11 (6.0)	23 (5.2)	9 (3.5)	4 (3.9)	4 (6.2)	51 (4.9)	
	Sometimes	28 (15.3)	77 (17.5)	47 (18.4)	18 (17.3)	16 (24.6)	186 (17.8)	
	Always	144 (78.7)	338 (76.8)	200 (78.1)	81 (77.9)	45 (69.2)	808 (77.1)	
	**Hand wash after toileting**							
	Never	0 (0.0)	1 (0.2)	0 (0.0)	0 (0.0)	0 (0.0)	1 (0.1)	0.443
	When needed	2 (1.1)	4 (0.9)	1 (0.4)	1 (1.0)	2 (3.1)	10 (1.0)	
	Sometimes	4 (2.2)	4 (0.9)	3 (1.2)	1 (1.0)	3 (4.6)	15 (1.4)	
	Always	177 (96.7)	431 (98.0)	252 (98.4)	102 (98.1)	60 (92.3)	1022 (97.5)	
	**Use of alcohol hard rub**							
	Never	6 (3.3)	26 (5.9)	12 (4.7)	4 (3.9)	5 (7.7)	53 (5.1)	0.812
	When needed	27 (14.8)	57 (13.0)	39 (15.2)	14 (13.5)	12 (18.5)	149 (14.2)	
	Sometimes	41 (22.4)	117 (26.6)	57 (22.3)	27 (26.0)	13 (20.0)	255 (24.3)	
	Always	109 (59.6)	240 (54.6)	148 (57.8)	59 (56.7)	35 (53.9)	591 (56.4)	
**Face Mask**	**Wear mask**							
	Never	0 (0.0)	2 (0.5)	1 (0.4)	1 (1.0)	0 (0.0)	4 (0.4)	0.599
	When needed	3 (1.6)	7 (1.6)	3 (1.2)	0 (0.0)	2 (3.1)	15 (1.4)	
	Sometimes	7 (3.8)	10 (2.3)	7 (2.7)	1 (1.0)	4 (6.2)	29 (2.8)	
	Always	173 (94.5)	421 (95.7)	245 (95.7)	102 (98.1)	59 (90.8)	1000 (95.4)	
**Household Hygiene**	**Put disinfectants into toilets**							
	Never	68 (37.2)	173 (39.3)	96 (37.5)	32 (30.8)	21 (32.3)	390 (37.2)	0.042*
	When needed	42 (23.0)	117 (26.6)	50 (19.5)	33 (31.7)	14 (21.5)	256 (24.4)	
	Sometimes	29 (15.9)	75 (17.1)	48 (18.8)	21 (20.2)	20 (30.8)	193 (18.4)	
	Always	44 (24.0)	75 (17.1)	62 (24.2)	18 (17.3)	10 (15.4)	209 (19.9)	
	**Put disinfectants into drain-pipe**							
	Never	58 (31.7)	160 (36.4)	100 (39.1)	37 (35.6)	20 (30.8)	375 (35.8)	0.108
	When needed	52 (28.4)	133 (30.2)	60 (23.4)	24 (23.1)	24 (36.9)	293 (28.0)	
	Sometimes	38 (20.8)	77 (17.5)	48 (18.8)	27 (26.0)	17 (26.2)	207 (19.8)	
	Always	35 (19.1)	70 (15.9)	48 (18.8)	16 (15.4)	4 (6.2)	173 (16.5)	
**Social Distancing**	**Avoid leaving home**							
	Never	7 (3.8)	15 (3.4)	9 (3.5)	1 (1.0)	4 (6.2)	36 (3.4)	0.947
	When needed	30 (16.4)	68 (15.5)	45 (17.6)	17 (16.4)	10 (15.4)	170 (16.2)	
	Sometimes	55 (30.1)	135 (30.7)	70 (27.3)	28 (26.9)	18 (27.7)	306 (29.2)	
	Always	91 (49.7)	222 (50.5)	132 (51.6)	58 (55.8)	33 (50.8)	536 (51.2)	
	**Avoid contacting neighbor**							
	Never	11 (6.0)	20 (4.6)	20 (7.8)	2 (1.9)	3 (4.6)	56 (5.3)	0.377
	When needed	21 (11.5)	57 (13.0)	29 (11.3)	12 (11.5)	11 (16.9)	130 (12.4)	
	Sometimes	35 (19.1)	82 (18.6)	41 (16.0)	26 (25.0)	16 (24.6)	200 (19.1)	
	Always	116 (63.4)	281 (63.9)	166 (64.8)	64 (61.5)	35 (53.9)	662 (63.2)	
**Availability of workplace guidelines and measures**								
	No	22 (12.0)	38 (8.6)	19 (7.4)	12 (11.5)	15 (23.1)	106 (10.1)	0.003**
	Yes	161 (88.0)	402 (91.4)	237 (92.6)	92 (88.5)	50 (76.9)	942 (89.9)	

*Note*: 1. Manual labourer: service/sales workers and craft workers, plant/machine operators and assemblers, elementary workers, and farm workers, animal husbandry workers and fishermen, occupations unidentifiable or inadequately described. 2. These P value came from Chi-square test or Fisher’s exact test

* *P* < 0.05; ** *P* < 0.01

### Personal protection behaviours for prevention of COVID-19

In Table 2, compliance with the four behavioural categories comprising eight personal protective behaviours was evaluated. Regarding hand hygiene, of the 1048 survey participants, 77.1% reported always washing hands before meals, and 97.5% reported always washing hands after using the toilet, but only 56.4% always used alcohol-based hand rub when outside. Regarding face mask use, 95.4% always wore a face mask when outside. As for household hygiene behaviours, only 19.9 and 16.5% always put disinfectant into toilets and drain-pipes, respectively. Regarding social distancing behaviours, 51.2% reported they always avoided leaving their home and 63.2% always avoided contact with their neighbours (Table 2).

### Compliance with personal protection behaviours by socio-demographic and occupation groups

In Table 1, personal protection behaviours differed between socio-demographic subgroups in terms of age, gender, living arrangement, marital status, educational level and work status. Participants aged below 50 years tended to wash hands more frequently after using the toilet (*P* < 0.05), while those under 30 years old washed hands less frequently before meals (P < 0.05). Females were more likely to always perform protection behaviours, especially using alcohol hand rub (61.9% vs. 44.6% for males, *P* < 0.001), avoiding leaving home (54.8% vs.43.5% for male, *P* < 0.05), and avoiding contacting neighbours (66.6% vs.56.0% for males, *P* < 0.05). The cohabiting participants showed a lower frequency of several behaviours than their single, married, divorced, and widowed counterparts, namely handwashing before meals (60.9%, P < 0.05), wearing mask (87.0%, P < 0.001), and avoiding contact with neighbours (43.5%, P < 0.001), while those who were single, divorced, or widowed reported a lower frequency of avoiding leaving home (*P* < 0.05). As for living arrangements, there was a clear pattern that participants living alone were less likely to perform protective behaviours than those living with others. In addition, there were no significant differences according to education attainment or work status for most of behaviours, except that those who finished higher secondary school reported lower frequency of using disinfectant in toilets (*P* < 0.05), and full-time employees were less likely to always avoid contact with neighbours (61.8% vs 76.5% of part-time employees, *P* < 0.05).

In Table 2, personal protection behaviour patterns in different occupations were mostly alike, except for manual laborers, who reported a slightly lower frequency of such behaviours. In the two-way cross tabulation analysis of occupation and frequency of behaviours (Table 2), a significant difference between occupations was only found for putting disinfectant into toilets (*P* < 0.05), with 24.0% of managers and administrators and 24.2% of associate professionals always performing this behaviour, and only 15.4% of manual laborers always doing so. For handwashing, approximately 78% of the combined professionals (i.e. managers and administrators, professionals, associated professionals, and clerical workers) always washed hands before meals, and around 98% of them always washed hands after using the toilet, while manual labourers reported slightly lower frequencies for these two behaviours (69.2 and 92.3% for handwashing before meals and after using toilet, respectively); however these, differences were not significant. Around 90.8% of manual labourers wore a face mask, while at least 94% individuals from the combined professionals always did so. As for social distancing, there was a lower proportion of manual labourers (53.9%) who always avoided contact with their neighbours in comparison with combined professionals (over 60%); however, these differences were not statistically significant.

### Inequality in workplace guideline and its relationship with personal protection behaviours

As shown in Tables 2, 89.9% of participants reported that relevant guidelines and measures were available in their workplace, while fewer manual laborers (76.9%) reported such availability (*P* = 0.003). The association between personal protection behaviours and availability of workplace guidelines and measures was examined (Table 3). After adjustment for covariates, individuals with available guidelines and measures in their workplace tended to report higher frequency of handwashing before meals (adjusted odds ratio [OR]: 4.21, 95% confidence interval [CI]: 1.62–10.95), handwashing after toilet use (adjusted OR: 7.54, 95% CI: 1.27–44.72), wearing a face mask (adjusted OR: 4.25, 95% CI: 1.02–17.76), and avoiding contact with neighbours (adjusted OR: 2.79, 95% CI: 1.15–6.76).

**Table 3:** Association of personal protection behaviours with workplace policy and socio-demographic factors

	Hand Hygiene			Face Mask	Household Hygiene		Social distancing	
	Hand wash before meals	Hand wash after toileting	Use alcohol hand rub	Wear mask	Put disinfectants into toilets	Put disinfectants into drain pipe	Avoid leaving home	Avoid contact neighbours
	OR (95% CI)	OR (95% CI)	OR (95% CI)	OR (95% CI)	OR (95% CI)	OR (95% CI)	OR (95% CI)	OR (95% CI)
**Workplace guideline and measures**								
Not available	Reference	Reference	Reference	Reference	Reference	Reference	Reference	Reference
Available	4.21** (1.62, 10.95)	7.54* (1.27, 44.72)	1.53 (0.65, 3.60)	4.25* (1.02, 17.76)	0.61 (0.27, 1.40)	0.58 (0.26, 1.30)	1.73 (0.73, 4.11)	2.79* (1.15, 6.76)
**Occupation**								
Manager and administrator	Reference	Reference	Reference	Reference	Reference	Reference	Reference	Reference
Professional	2.52 (0.82, 7.73)	3.00 (0.26, 34.24)	1.10 (0.40, 3.05)	1.56 (0.29, 8.46)	0.46 (0.17, 1.22)	0.51 (0.20, 1.33)	1.36 (0.50, 3.70)	2.00 (0.69, 5.78)
Associate professional	8.31* (1.49, 46.47)	–	1.41 (0.40, 4.92)	–	0.78 (0.24, 2.53)	0.97 (0.30, 3.10)	2.96 (0.85, 10.34)	3.95* (1.05, 14.91)
Clerical support worker	2.28 (0.50, 10.45)	0.81 (0.05, 12.90)	1.43 (0.36, 5.71)	1.79 (0.13, 24.82)	0.49 (0.14, 1.70)	0.33 (0.09, 1.25)	2.53 (0.58, 11.16)	1.92 (0.44, 8.49)
Manual labourer	0.83 (0.22, 3.08)	0.90 (0.10, 8.19)	0.44 (0.13, 1.51)	0.52 (0.08, 3.23)	0.38 (0.11, 1.33)	0.24 (0.07, 0.84)	0.30 (0.08, 1.12)	0.56 (0.16, 2.03)
**Interaction: workplace guideline x occupation**								
Available x. Professional	0.35 (0.10, 1.16)	0.22 (0.01, 3.69)	0.67 (0.23, 1.97)	0.51 (0.07, 3.56)	1.83 (0.65, 5.11)	1.68 (0.62, 4.60)	0.75 (0.26, 2.15)	0.43 (0.14, 1.33)
Available x Associate Professional	0.11* (0.02, 0.65)	–	0.56 (0.15, 2.09)	–	1.37 (0.40, 4.71)	0.88 (0.26, 2.98)	0.33 (0.09, 1.23)	0.22* (0.05, 0.88)
Available x Clerical support Workers	0.42 (0.08, 2.17)	1.67 (0.05, 58.42)	0.54 (0.13, 2.33)	1.65 (0.06, 48.25)	1.97 (0.53, 7.27)	3.14 (0.77, 12.72)	0.44 (0.09, 2.05)	0.42 (0.09, 2.02)
Available x Manual labourer	1.06 (0.23, 4.79)	0.27 (0.02, 4.39)	1.81 (0.46, 7.17)	0.84 (0.08, 8.42)	3.10 (0.79, 12.25)	3.81 (1.00, 14.53)	4.43* (1.06, 18.47)	1.21 (0.30, 4.92)
**Age group**								
18–29	Reference	Reference	Reference	Reference	Reference	Reference	Reference	Reference
30–39	1.43 (0.92, 2.23)	4.29 (0.70, 26.17)	1.03 (0.71, 1.49)	0.93 (0.34, 2.54)	1.12 (0.79, 1.58)	1.05 (0.74, 1.49)	1.23 (0.85, 1.77)	0.97 (0.66, 1.44)
40–49	1.29 (0.80, 2.08)	1.88 (0.41, 8.61)	1.05 (0.70, 1.56)	0.89 (0.31, 2.53)	1.41 (0.97, 2.04)	1.27 (0.88, 1.85)	1.17 (0.79, 1.73)	0.98 (0.65, 1.50)
50–59	1.17 (0.67, 2.07)	0.60 (0.13, 2.76)	0.70 (0.44, 1.11)	0.70 (0.22, 2.24)	1.23 (0.79, 1.92)	1.02 (0.66, 1.59)	1.06 (0.67, 1.66)	0.83 (0.51, 1.34)
60+	2.80 (0.84, 9.41)	0.62 (0.08, 5.04)	0.96 (0.41, 2.22)	0.48 (0.09, 2.44)	1.77 (0.83, 3.76)	1.72 (0.80, 3.70)	1.87 (0.81, 4.32)	1.18 (0.49, 2.85)
**Gender**								
Female	Reference	Reference	Reference	Reference	Reference	Reference	Reference	Reference
Male	0.71* (0.52, 0.98)	1.22 (0.49, 2.99)	0.45* (0.35, 0.59)	0.66 (0.35, 1.24)	0.75* (0.59, 0.96)	0.92 (0.72, 1.17)	0.60* (0.47, 0.78)	0.66** (0.51, 0.87)
**Marital status**								
Never married	Reference	Reference	Reference	Reference	Reference	Reference	Reference	Reference
Married	1.65* (1.12, 2.44)	0.38 (0.10, 1.35)	1.28 (0.93, 1.74)	0.93 (0.41, 2.11)	0.84 (0.63, 1.13)	0.91 (0.69, 1.22)	1.31 (0.97, 1.77)	1.19 (0.87, 1.64)
Cohabited	0.74 (0.27, 2.06)	0.09 (0.01, 1.04)	0.77 (0.30, 1.97)	0.32 (0.06, 1.72)	0.70 (0.30, 1.62)	0.81 (0.35, 1.90)	0.96 (0.38, 2.44)	0.45 (0.17, 1.17)
Divorced/ separated	1.80 (0.74, 4.38)	1.15 (0.12, 11.54)	1.53 (0.76, 3.07)	2.79 (0.33, 23.38)	1.10 (0.56, 2.17)	1.40 (0.75, 2.62)	1.03 (0.52, 2.02)	1.50 (0.70, 3.22)
Widowed	1.24 (0.21, 7.36)	0.29 (0.02, 5.40)	0.69 (0.17, 2.81)	0.51 (0.04, 5.83)	2.58 (0.69, 9.60)	2.51 (0.63, 10.06)	0.54 (0.16, 1.87)	0.30 (0.08, 1.13)
**Living arrangement**								
Alone	Reference	Reference	Reference	Reference	Reference	Reference	Reference	Reference
With family	1.29 (0.70, 2.39)	6.27** (1.57, 25.00)	1.46 (0.87, 2.45)	3.40* (1.27, 9.09)	2.30** (1.36, 3.88)	2.12** (1.26, 3.56)	1.04 (0.62, 1.75)	1.20 (0.69, 2.08)
With others	1.17 (0.43, 3.22)	–	2.04 (0.85, 4.87)	2.17 (0.36, 13.17)	4.59* (2.02, 10.45)	2.26 (1.00, 5.11)	1.72 (0.70, 4.23)	2.16 (0.80, 5.80)
**Education attainment**								
Lower secondary school	Reference	Reference	Reference	Reference	Reference	Reference	Reference	Reference
Higher secondary school	2.72 (0.76, 9.75)	13.16* (1.32, 131.35)	0.85 (0.24, 3.02)	1.46 (0.12, 17.36)	0.37 (0.11, 1.21)	0.49 (0.14, 1.66)	1.17 (0.34, 4.03)	1.14 (0.29, 4.56)
Preparatory course	3.51 (0.98, 12.57)	5.74 (0.66, 50.01)	1.07 (0.30, 3.82)	1.68 (0.14, 19.87)	0.37 (0.12, 1.21)	0.56 (0.17, 1.89)	0.96 (0.28, 3.30)	0.83 (0.21, 3.29)
Undergraduate or above	3.08 (0.89, 10.65)	6.81 (0.89, 52.30)	0.97 (0.28, 3.39)	1.23 (0.11, 13.41)	0.39 (0.12, 1.24)	0.51 (0.15, 1.69)	1.15 (0.34, 3.88)	1.03 (0.26, 3.99)
**Work status**								
Full-time	Reference	Reference	Reference	Reference	Reference	Reference	Reference	Reference
Part-time	0.93 (0.54, 1.60)	2.74 (0.53, 14.19)	1.03 (0.65, 1.62)	1.27 (0.40, 3.98)	1.02 (0.68, 1.54)	0.82 (0.54, 1.26)	1.42 (0.90, 2.26)	2.02* (1.18, 3.47)

*Note*: 1. Manual labourer: service/sales workers and craft workers, plant/machine operators and assemblers, elementary workers, and farm workers, animal husbandry workers and fishermen, occupations unidentifiable or inadequately described

* *P* < 0.05; The values reported in the table are adjusted odds ratios and their 95% Confidence intervals (CI)

The significant ORs for the interaction terms of workplace policy availability and occupation presented in Table 3 suggest that the association between workplace policy availability and personal protection behaviours was different between occupational groups. The association was also examined for different occupation groups separately (Table 4). The association between workplace policy availability and personal protection behaviours was found to be significant for managers and administrators, as well as manual labourers, with adjustment for covariates, while no significant association was found for other occupations. Among managers and administrators, the availability of workplace guidelines was found to be associated with handwashing before meals (adjusted OR: 4.56, 95% CI: 1.64–12.63), wearing a face mask (adjusted OR: 9.52, 95% CI: 1.79–50.53), avoiding leaving home (adjusted OR: 2.73, 95%CI: 1.04–7.16), and avoiding contact with neighbours (adjusted OR: 4.98, 95% CI: 1.81–13.72). Among manual laborers, the availability of guidelines was associated with handwashing before meals (adjusted OR: 4.76, 95% CI: 1.10–20.69), using alcohol-based hand rub for disinfection (adjusted OR: 6.21, 95% CI: 1.54–25.12), avoiding leaving home (adjusted OR: 9.42, 95% CI: 2.14–41.56), and avoiding contact with neighbours (adjusted OR: 4.58, 95% CI: 1.09–19.22). Although a significant association was found, the standard errors were relatively large, and 95% CIs were also wide because of a relatively small sample size for a few behaviours and occupational groups; thus, the interpretation of point estimates of adjusted ORs should take this into consideration.

**Table 4:** Association of personal protection behaviours with workplace policy among different occupation groups

		Availability of workplace guideline and measures
		Adjusted OR^**1**^ (95% CI)
**Hand Hygiene**	**Hand wash before meals**	
	Manager	4.56** (1.64, 12.63)
	Professional	1.13 (0.52, 2.48)
	Associate professional	0.33 (0.06, 1.70)
	Clerical workers	6.09 (0.84, 44.40)
	Manual labourer^2^	4.76* (1.10, 20.69)
	**Hand wash after toileting**	
	Manager	9.35 (0.40, 216.31)
	Professional	1.41 (0.11, 17.90)
	Associate professional	---^3^
	Clerical workers	---^3^
	Manual labourer	---^3^
	**Use of alcohol hand rub**	
	Manager	1.53 (0.60, 3.91)
	Professional	1.09 (0.55, 2.17)
	Associate professional	0.77 (0.26, 2.24)
	Clerical workers	0.36 (0.08, 1.75)
	Manual labourer	6.21* (1.54, 25.12)
**Face Mask**	**Wear Mask**	
	Manager	9.52** (1.79, 50.53)
	Professional	2.00 (0.47, 8.55)
	Associate professional	---^3^
	Clerical workers	---^3^
	Manual labourer	3.49 (0.24, 51.32)
**Household Hygiene**	**Put disinfectants into toilets**	
	Manager	0.68 (0.28, 1.65)
	Professional	1.03 (0.55, 1.95)
	Associate professional	0.71 (0.27, 1.86)
	Clerical workers	2.39 (0.70, 8.10)
	Manual labourer	3.46 (0.86, 14.00)
	**Put disinfectants into drain-pipe**	
	Manager	0.69 (0.29, 1.63)
	Professional	0.85 (0.45, 1.60)
	Associate professional	0.47 (0.19, 1.21)
	Clerical workers	2.42 (0.62, 9.50)
	Manual labourer	2.91 (0.76, 11.17)
**Social Distancing**	**Avoid leaving home**	
	Manager	2.73* (1.04, 7.16)
	Professional	1.37 (0.72, 2.58)
	Associate professional	0.63 (0.22, 1.77)
	Clerical workers	1.57 (0.33, 7.40)
	Manual labourer	9.42** (2.14, 41.56)
	**Avoid contacting neighbour**	
	Manager	4.98** (1.81, 13.72)
	Professional	1.21 (0.59, 2.51)
	Associate professional	0.55 (0.18, 1.71)
	Clerical workers	2.34 (0.45, 12.21)
	Manual labourer	4.58* (1.09, 19.22)

*Note*: 1. Adjusted ORs in this table were estimated in separate ordinal logistic regression models of personal protection behaviours with adjustment of covariates for corresponding occupation group, i.e. the estimates and confidence interval presented in this table all came from different models for different occupation groups where behaviour was dependent variable and workplace policy and other factors were independent variables. 2. Manual labourer: service/sales workers and craft workers, plant/machine operators and assemblers, elementary workers, and farm workers, animal husbandry workers and fishermen, occupations unidentifiable or inadequately described. 3. These estimates and confidence intervals were omitted in their regression models due to non-convergence

* *P* < 0.05; ** *P* < 0.01

### Sensitivity analysis: relationship between personal protection behaviours and workplace guidelines in a weighted sample

In the weighted sample, there were 121 manager and administrators, 82 professionals, 216 associate professionals, 134 clerical support workers and 493 manual labourers. The occupation distribution in the weighted sample is consistent with the entire HK working population. The results of multiple ordinal logistic regression are shown in Table 5. Individuals with relevant workplace guidelines for COVID-19 prevention in the workplace were more likely to have a higher frequency of handwashing before meals (adjusted OR: 4.25, 95% CI: 1.29–14.06) and handwashing after toilet use (adjusted OR: 18.79, 95% CI: 1.65–213.54). The association was also marginally significant in avoiding contact with neighbours (adjusted OR: 3.04, 95% CI: 0.98–9.41). Association of wearing face mask with workplace guidelines, which was significant in the original study sample, showed a similar point estimate but a larger confidence interval (adjusted OR: 4.40, 95% CI: 0.75–25.84) compared with the estimates from the original sample. In general, the primary findings shown in the weighted sample are similar to those in the original survey sample.

**Table 5:** Sensitivity analysis: association of personal protection behaviours with workplace policy and socio-demographic factors in a weighted sample

	Hand Hygiene			Face Mask	Household Hygiene		Social distancing	
	Hand wash before meals	Hand wash after toileting	Use alcohol hand rub	Wear mask	Put disinfectants in toilets	Put disinfectants in drain pipe	Avoid leaving home	Avoid contact neighbours
	OR (95% CI)	OR (95% CI)	OR (95% CI)	OR (95% CI)	OR (95% CI)	OR (95% CI)	OR (95% CI)	OR (95% CI)
**Workplace guideline and measures**								
Not available	Reference	Reference	Reference	Reference	Reference	Reference	Reference	Reference
Available	4.25* (1.29, 14.06)	18.79* (1.65, 213.54)	1.53 (0.53, 4.43)	4.40 (0.75, 25.84)	0.63 (0.22, 1.82)	0.49 (0.18, 1.32)	1.80 (0.63, 5.09)	3.04 (0.98, 9.41)
**Occupation**								
Manager and administrator	Reference	Reference	Reference	Reference	Reference	Reference	Reference	Reference
Professional	2.47 (0.35, 17.57)	4.23 (0.02, 880.55)	1.04 (0.17, 6.22)	1.39 (0.05, 37.31)	0.39 (0.07, 2.20)	0.34 (0.06, 1.88)	1.27 (0.24, 6.78)	1.97 (0.29, 13.27)
Associate professional	7.67* (1.08, 54.32)	–	1.36 (0.32, 5.75)	–	1.02 (0.25, 4.19)	0.92 (0.23, 3.69)	2.70 (0.65, 11.21)	5.64* (1.17, 27.08)
Clerical support worker	2.11 (0.45, 9.89)	0.44 (0.03, 6.53)	1.35 (0.33, 5.53)	0.49 (0.04, 5.72)	0.22* (0.06, 0.82)	0.20* (0.05, 0.78)	2.58 (0.59, 11.28)	2.10 (0.46, 9.63)
Manual labourer^1^	0.75 (0.23, 2.39)	0.76 (0.12, 5.07)	0.42 (0.14, 1.21)	0.45 (0.09, 2.22)	0.27* (0.09, 0.79)	0.20** (0.07, 0.56)	0.30* (0.10, 0.86)	0.64 (0.21, 1.99)
**Interaction: workplace guideline x occupation**								
Available x. Professional	0.36 (0.04, 2.90)	0.08 (0, 28.5)	0.68 (0.10, 4.44)	0.49 (0.01, 18.44)	2.41 (0.39, 14.85)	2.50 (0.42, 14.91)	0.79 (0.13, 4.60)	0.43 (0.06, 3.17)
Available x Associate Professional	0.12* (0.02, 0.92)	–	0.61 (0.13, 2.80)	–	1.30 (0.29, 5.74)	0.90 (0.21, 3.85)	0.35 (0.08, 1.54)	**0.15** (0.03, 0.79)
Available x Clerical support Workers	0.45 (0.08, 2.44)	1.35 (0.04, 49.05)	0.55 (0.12, 2.47)	4.66 (0.19, 113.82)	4.26* (1.06, 17.18)	5.20* (1.20, 22.42)	0.40 (0.08, 1.88)	0.42 (0.08, 2.10)
Available x Manual labourer	1.17 (0.32, 4.20)	0.09 (0.01, 1.14)	1.95 (0.62, 6.10)	0.76 (0.11, 5.22)	3.69* (1.17, 11.58)	4.36* (1.47, 12.93)	4.11* (1.33, 12.76)	1.23 (0.37, 4.13)
**Age group**								
18–29	Reference	Reference	Reference	Reference	Reference	Reference	Reference	Reference
30–39	1.61 (0.98, 2.63)	2.61 (0.24, 28.37)	1.32 (0.86, 2.02)	0.74 (0.21, 2.55)	2.18** (1.46, 3.26)	1.75** (1.19, 2.58)	1.27 (0.84, 1.91)	0.76 (0.49, 1.16)
40–49	1.37 (0.79, 2.36)	0.80 (0.11, 5.77)	1.14 (0.72, 1.81)	1.43 (0.38, 5.35)	2.90** (1.87, 4.49)	1.52 (1.00, 2.32)	1.15 (0.74, 1.81)	1.24 (0.76, 2.03)
50–59	1.11 (0.62, 1.99)	0.05** (0.01, 0.39)	0.76 (0.47, 1.25)	0.27* (0.08, 0.93)	2.55** (1.59, 4.09)	1.13 (0.71, 1.80)	0.71 (0.44, 1.15)	0.49** (0.29, 0.82)
60+	2.30 (0.94, 5.61)	0.04** (0.01, 0.37)	1.26 (0.62, 2.56)	0.12* (0.03, 0.51)	2.53** (1.36, 4.69)	0.98 (0.52, 1.83)	2.02 (1.00, 4.09)	1.84 (0.83, 4.08)
**Gender**								
Female	Reference	Reference	Reference	Reference	Reference	Reference	Reference	Reference
Male	0.73* (0.53, 1.02)	3.56** (1.37, 9.25)	0.49** (0.37, 0.64)	0.98 (0.53, 1.80)	0.64** (0.50, 0.83)	0.77* (0.60, 0.99)	0.59** (0.45, 0.77)	0.49** (0.37, 0.64)
**Marital status**								
Never married	Reference	Reference	Reference	Reference	Reference	Reference	Reference	Reference
Married	1.91** (1.22, 2.98)	0.28 (0.07, 1.15)	1.94** (1.35, 2.79)	0.58 (0.23, 1.49)	0.94 (0.67, 1.31)	1.05 (0.76, 1.45)	1.37 (0.97, 1.93)	1.18 (0.82, 1.70)
Cohabited	0.43 (0.16, 1.15)	0.21 (0, 32.37)	0.32* (0.13, 0.81)	1.12 (0.07, 19.07)	1.07 (0.47, 2.43)	1.23 (0.54, 2.80)	2.00 (0.70, 5.67)	0.34* (0.14, 0.84)
Divorced/ separated	2.57** (1.27, 5.21)	0.22 (0.05, 1.02)	0.68 (0.40, 1.17)	0.55 (0.17, 1.81)	1.93* (1.15, 3.24)	2.09** (1.27, 3.45)	1.66 (0.97, 2.86)	1.37 (0.79, 2.38)
Widowed	1.44 (0.21, 9.88)	0.26 (0.01, 6.22)	1.05 (0.23, 4.70)	0.37 (0.03, 5.18)	1.76 (0.38, 8.11)	2.98 (0.60, 14.73)	0.72 (0.19, 2.76)	0.19* (0.04, 0.86)
**Living arrangement**								
Alone	Reference	Reference	Reference	Reference	Reference	Reference	Reference	Reference
With family	1.14 (0.53, 2.46)	6.17* (1.29, 29.54)	2.17* (1.15, 4.11)	2.93* (0.86, 9.93)	2.67** (1.46, 4.91)	2.87** (1.53, 5.40)	1.43 (0.78, 2.64)	1.02 (0.51, 2.05)
With others	1.56 (0.43, 5.69)	–	8.28** (2.67, 25.68)	0.58 (0.05, 6.74)	5.31** (1.99, 14.15)	2.06 (0.81, 5.26)	1.47 (0.50, 4.31)	1.60 (0.49, 5.20)
**Education attainment**								
Lower secondary school	Reference	Reference	Reference	Reference	Reference	Reference	Reference	Reference
Higher secondary school	4.60** (2.36, 8.98)	31.33** (7.83, 125.38)	1.84 (0.99, 3.41)	8.53** (2.48, 29.37)	1.45 (0.76, 2.79)	3.16** (1.64, 6.07)	2.84** (1.53, 5.29)	2.52** (1.29, 4.92)
Preparatory course	7.80** (3.80, 16.05)	21.24** (3.88, 116.13)	2.95** (1.53, 5.67)	4.97** (1.53, 16.14)	0.54 (0.27, 1.06)	3.52** (1.8, 6.87)	3.13** (1.63, 6.01)	1.90 (0.96, 3.73)
Undergraduate or above	5.02** (2.54, 9.90)	3.28* (1.02, 10.63)	2.38** (1.25, 4.51)	2.68 (0.89, 8.13)	0.92 (0.47, 1.79)	2.80** (1.45, 5.43)	2.47** (1.31, 4.66)	2.91** (1.49, 5.71)
**Work status**								
Full-time	Reference	Reference	Reference	Reference	Reference	Reference	Reference	Reference
Part-time	1.08 (0.69, 1.68)	3.32* (1.06, 10.43)	1.73** (1.17, 2.55)	4.32** (1.53, 12.17)	2.91** (2.06, 4.12)	1.41* (1.01, 1.99)	1.51* (1.04, 2.20)	2.97** (1.91, 4.62)

*Note*: 1. Manual labourer: service/sales workers and craft workers, plant/machine operators and assemblers, elementary workers, and farm workers, animal husbandry workers and fishermen, occupations unidentifiable or inadequately described

* *P* < 0.05; ** *P* < 0.01; The values reported in the table are adjusted odds ratios and their 95% Confidence intervals (CI)

## Discussion

During the current COVID-19 pandemic, few studies so far have looked at personal protection behaviours and their relationship with the availability of workplace policy related to COVID-19 prevention. In considering workplace policy availability during the COVID-19 pandemic, this study revealed that manual laborers had a significantly lower availability of workplace policy and relatively lower compliance of selected personal protection behaviours than those with other occupations. This inequality should receive greater attentions by government, employers and employees in further prevention practice.

For personal protection behaviours, most employees performed hand hygiene and wore face masks, and over half of them showed high compliance with social distancing. Maintaining household hygiene was measured as frequency of using disinfection for toilets and drain-pipes, which comes from the experience of the2003 Severe Acute Respiratory Syndrome (SARS) outbreaks in HK, where the virus was likely to be transmitted through sanitary plumbing systems [23, 24]. However, these behaviours were found to be performed by fewer participants in this study, which might be related to the fact that no cases of COVID-19 infection have been confirmed to occur through buildings’ plumbing systems locally at the time of the present survey. Another possible reason for the low compliance with household hygiene might be related to the difficulty in accessing detergents in the supermarket or online [25].

Our findings also indicated that individuals living alone showed a lower frequency of protection behaviours, which highlights the importance of social support from the family in promoting prevention at the individual level, as those living alone might have lower the accessibility to relevant information, awareness, and concern about performing such behaviours because of a lack of reminders from other sources. In light of this, governments and local communities should be able to approach these individuals to promote community-level health education and information dissemination.

This study found that not only employees with different occupations showed differences in personal protection behaviours, but workplace policy availability was also a factor related to such behaviours. The employees with access to guidelines and measures for the prevention of COVID-19 showed a higher frequencies of protection behaviours, especially hand hygiene, wearing a face mask, and social distancing. This indicated that publishing guidelines and implementing prevention measures in the workplace is associated with greater compliance of the personal protection behaviours of employees.

Regarding the relationship between workplace policy availability and personal protection behaviours, only managers/administrators and manual labourers who had access to guidelines and measures in their workplace were more likely to perform adequate protection behaviours. On one hand, managers and administrators might be the persons who have the authority to make decisions about providing such guidelines and measures in their workplace. Therefore, their own frequency of protection behaviours could reflect their knowledge, awareness, and attitude regarding the prevention of COVID-19, and this might consequently affect the availability of guidelines and measures in their workplace. On the other hand, the lower adoption and compliance of a few personal protection behaviours among manual labourers, including hand hygiene and social distancing, was possibly related to inadequate workplace policies. This association was not significant for behaviours related to household hygiene, implying that the workplace policies may not improve the compliance of personal protection behaviours that are typically performed at home, even though they were associated with other precautions during their work and daily life. As mentioned earlier, most manual laborers come from lower socio-economic backgrounds and experience worse working conditions, such as longer working hours and heavier working load, than other occupational groups; this could hinder their access to adequate knowledge and awareness of the need for such protection behaviours [26]. The nature of their work also requires them to make contact with other individuals, especially for those working in sales and service provision, so it could be difficult for them to keep social distance from the crowds if no special work arrangements are put in place for them [27, 28]. Moreover, a supplementary analysis revealed that the information transparency on COVID-19 infection among co-workers was much lower among manual labourers without workplace policies for COVID-19 prevention (see Additional file 1: Table S3). Thus, this finding suggests that workplace guidelines and measures may serve as an important way to communicate relevant information to manual laborers to increase their awareness and attention and encourage them to comply with the health behaviours. Additionally, it could also be difficult for these workers to obtain hand disinfectants and face masks due to the shortage of supplies and inflated prices. For manual labourers, available workplace policies for COVID-19 prevention were also associated with greater accessibility to adequate personal protection equipment (PPE) and products that were provided by their employers (see Additional file 1: Table S1-S2), which could increase their compliance with health behaviours. Meanwhile, other occupations such as professionals might have more knowledge and information about COVID-19 and its prevention; thus, workplace policy availability did not have a significant association with their behaviours.

Our findings, which suggest that it is important to promote the prevention of COVID-19 at the individual level by improving workplace guidelines and measures, can be used to inform formulation of workplace policies and interventions to prevent spread of COVID-19. Individual and organizational level policies for workplace preventions should primarily target to the managers and administrators and manual labourers based on the study results. On one hand, improvements are needed for employers as well as managers and administrators to enhance the availability of guidelines; moreover, there should be instructions or templates to develop guidelines and measures provided by health authorities as a reference for administrators to follow and develop their own version. Adequate training for infection control can be made available to employees to improve their awareness and hygiene practice based on experiences from outbreaks and epidemics of other diseases [22, 29]. On the other hand, among the different occupations, more efforts are required to provide workplace prevention guidelines and measures for manual labourers, including service workers/sales/craft workers, plant/machine operators, and assemblers and elementary workers, as their reported availability of such guidelines was lower, and some of them, such as service/sales workers, are exposed to a higher risk than others because of having more frequent contact with other individuals. According to the results (Table 4), guidelines which acts as a reminder or encouragement to the manual labourers should be provided to increase their awareness of hand hygiene and social distancing restrictions. Moreover, availability and transparency of COVID-19 related information could also increase the mutual understanding between employer and employee. It therefore enhances the compliance of their health behaviours. This finding is supported by a China study that identified exposure to COVID-19 information was associated with higher compliance of wearing masks and hand sanitizing among factory workers [30]. The effectiveness of specific guidelines and measures for them to improve their personal protection behaviours can be further examined in future studies.

Previous research showed the governments played an important role in promoting workplace policies for COVID-19 prevention, thus a swift and coordinated policy at regional and national level with a strong multilateral leadership is required. Mainland China extended public holidays and issued a mandatory work-from-home policy for inessential positions in late January and early February 2020 [31]. The government of Japan and Singapore have encouraged employees to take leave if they have symptoms and promoted teleworking and staggered office hours [7]. Singapore closed all the workplaces for non-essential services in April 2020 [32]. The HK government has also implemented work-from-home arrangements for government employees since February and urged the private sector to make flexible work arrangements for employees in late March 2020 [33]. Although the workplace policies of the infection control had at least moderate effects in reducing COVID-19 attacks [34] by reducing the frequencies of contacts and ensuring social distancing, it may not be applicable for all the manual labourers. It is because most of the manual labourers cannot work from home due to the nature of their work, and they could easily lose their jobs due to workplace closure and COVID-19 related transportation restrictions [35, 36]. Besides the workplace closure and work-from-home policies during the pandemic, a wider range of workplace policies suggested to be decide to employees. In order to avoid lack of protection due to shortage of the equipment, adequate PPE for COVID-19 prevention should be made available by the employers at workplace with the support of governments, including hand sanitizer and face masks. Singapore government acts as an example that the employers should ensure there are sufficient masks for employees [37]. Mandatory mask wearing at workplace can be considered where people are close to each other in working areas. Mainland China [38] and Singapore were the few countries launching the related policy since early 2020 [37]. Employees also demand the related instructions on how to properly maintain hygiene and wear a face mask [19]. This information could help improve the adequacy and effectiveness of their protection behaviours. These measures that are universally available to all workplaces are expected to improve the equalities of accessibility of materials, information, and workplace guidelines for COVID-19 preventions across occupations. In addition, more attentions should be paid to vulnerable working population. Some individuals had concerns about salary penalties for absence due to influenza or COVID-19 [19]. This kind of concern may reduce employees’ willingness to comply with suggestions such as staying at home and keeping social distance if they experience symptoms, especially for employees with lower income, which would consequently expose their colleagues and general public to a higher risk. Therefore, paid sick leave, medical benefits, and/or insurance for compensating the income loss due to COVID-19 infection can be provided to financially vulnerable workers, especially for essential workers who need to stay in work position during the pandemics [39].

### Limitation

First, the survey adopted a convenience sampling method, which is not a parametric sampling method and there was no sampling frame. The sample collected might not represent the working population in HK well. Indeed, the study sample comprised more professionals and managers/ administrators, and fewer manual laborers compared with the occupational distribution of the employed population in HK. This is probably because the online questionnaire for participants were distributed through the social networking platforms of the research team and the institution in the first place, whose primary audiences are professionals. Therefore, there might have been selection bias due to the recruitment method. On one hand, those who participated into this survey might have greater concerns over the epidemic/pandemic situation, and therefore, they might have greater awareness and compliance with personal protection behaviour than those who did not participate. The compliance level of some personal protection behaviours in the general working population might not be as high as what was found in this study sample, such as mask wearing or hand-washing. On the other hand, the relationship between workplace policy and employee’s personal protection behaviours could be different according to their occupation (i.e. occupation is an effect modifier). To minimize the influence of the disproportionate occupation distribution in our sample for the regression findings, two additional analysis were performed. The first of these was performed in different occupations separately to reflect the differences between occupations to ensure that this results would not be affected by distribution of occupations. The second additional analysis was a sensitivity analysis using a sample adjusted by occupations, which means participants of different occupations were weighted to have an accurate occupation distribution of the working population in HK. The extra findings showed that the disproportionate occupation distribution did not have a substantial influence on the primary finding of this study. Nevertheless, more robust sampling, recruitment and data collection methods should be adopted in future studies.

Second, the number of participants in some occupational groups and with certain experience of workplace policy availability was relatively small, which led to larger standard errors of adjusted OR estimates or non-convergence of statistical models. Therefore, the estimates of the regression models were reported and interpreted along with their confidence intervals, which reflect their standard errors. Third, “when needed” and “sometimes” could be similar in describing the frequencies of the behaviours. “When needed” was used to indicate that individuals would perform the behaviours only when they believed it necessary, while “sometimes” was used to indicate that the individuals would comply with the behaviours from time to time. In addition, the type and contents of workplace guidelines and measures were not specified in the survey; therefore, conclusions on whether a specific measure or workplace policy may be associated with personal protection behaviours need to be drawn in future studies.

## Conclusion

The COVID-19 pandemic not only affects the global health, but also transforms into an economic and labour market shock. Protecting workers and their families from the infectious disease outbreak needs to be a top priority. This study provided evidence on current flaws in workplace policy availability regarding COVID-19 prevention and its inequality across occupations, as well as its association with personal protection behaviours. Attention is needed from multiple parties, including employers, employees, and governments, to increase awareness about the importance of workplace guidelines and measures in preventing COVID-19 spread at individual and community levels, especially for manual labourers, given that adequate workplace policy was associated with greater frequencies of employees’ behaviours for preventing COVID-19 spread. The workplace policies should include not only workplace closure and work-from-home arrangements, but also a wider range of responses and guidelines to ensure accessibility to infection control materials and information for essential workers, who have to attend to their workplace, and financially vulnerable workers cannot afford to be absence of work. The workplace policy may also serve as a way to disseminate information for health promotion and education regarding COVID-19; therefore, more efforts and resources need to be invested in this area by public health authorities. Further studies are also needed in different regions and countries for a thorough evaluation of workplace policies.

## Supplementary Information

**Additional file 1 Table S1.** Association between availability of workplace policies and supply of PPE by occupations. **Table S2.** Association between availability of workplace policies and adequacy of workplace PPE storage by occupations. **Table S3.** Association between availability of workplace policies and information transparency on the infections occurred among co-workers and their family by occupations.

## Data Availability

Data used in this study cannot be made publicly available for ethical reasons. Public availability of data would compromise confidentiality and privacy of participants.

## Abbreviations

CDC: Centre for Disease Control and Prevention
COVID-19: Coronavirus disease 2019
EU-OSHA: European Agency for Safety and Health at Work
SARS: Severe acute respiratory syndrome
SARS-CoV-2: Severe acute respiratory syndrome coronavirus 2
WHO: World Health Organization
