# Supplementary material for: Unequal availability of workplace policy for prevention of coronavirus disease 2019 across occupations and its relationship with personal protection behaviours: a cross-sectional survey
Source: Int J Equity Health. 2021 Sep 7;20:200. doi: 10.1186/s12939-021-01527-x (PMC8422060; doi:10.1186/s12939-021-01527-x)
Supplement: Supplementary file 1 — Additional file 1 Table S1. Association between availability of workplace policies and supply of PPE by occupations. Table S2. Association between availability of workplace policies and adequacy of workplace PPE storage by occupations. Table S3. Association between availability of workplace policies and information transparency on the infections occurred among co-workers and their family by occupations. [file 12939_2021_1527_MOESM1_ESM.docx]

**Supplemental analysis**

Additional analysis was performed to explore the relationship between availability of workplace policies and supply of personal protection equipment (PPE), adequacy of workplace PPE storage, and information transparency on COVID-19 infections among co-workers in different occupations, which aimed to look for potential explanations for the differences in association between availability of workplace policies and personal protection behaviours across occupations.

The results can be found in Table S1-S3. In Table S1, it can be found that manual labourers (74.0% vs 40.0%, P<0.05) and clerical workers (76.1% vs 33.3%, P<0.01) with workplace policies for COVID-19 prevention were more likely to be offered with PPE by employers, while this difference was not significant among other occupations. In Table S2, the gaps of adequacy of workplace PPE storage between those with workplace policies and without the policies were only significant among manual labourers (44.1% vs 7.7%, P<0.05). In Table S3, a similar pattern was found that the difference of information transparency on workplace-related COVID-19 information between those with workplace policies and without the policies were larger in manual labourers (70.4% vs 16.7%, P<0.01) than other occupations.

In general, for manual laborers, if their employers do not provide policies for COVID-19 prevention at workplace, they were also less likely to have adequate accessibility to the PPE and have sufficient transparency of information, which could enable and encourage them to commit to the personal protection behaviours. However, for other occupations, the gaps of accessibility of PPE and COVID-19 related information at workplace were smaller between those with and without such workplace policies. It was probably because employers of people with these occupations might have routinely available mechanism to share relevant information of the emergencies and provide support to their employees, while this mechanism might be limited in the workplace of manual labourers. Therefore, workplace policies that are designated for COVID-19 prevention might enable the establishment of workplace response mechanism for manual labourers.

Table S1. Association between availability of workplace policies and supply of PPE by occupations

| Occupation | Availability of workplace policies for COVID-19 prevention | | | | |
| --- | --- | --- | --- | --- | --- |
|  |  | Not available | Available | Total | P value |
|  | Supply of PPE^ by employers | | | |  |
| Administrator and manager | No | 11 (50.0) | 49 (30.4) | 60 (32.8) | 0.067 |
|  | Yes | 11 (50.0) | 112 (69.6) | 123 (67.2) |  |
|  | Total | 22 (100.0) | 161 (100.0) | 183 (100.0) |  |
|  |  |  |  |  |  |
| Professionals | No | 20 (52.6) | 148 (36.8) | 168 (38.2) | 0.055 |
|  | Yes | 18 (47.4) | 254 (63.2) | 272 (61.8) |  |
|  | Total | 38 (100.0) | 402 (100.0) | 440 (100.0) |  |
|  |  |  |  |  |  |
| Associate professionals | No | 8 (42.1) | 58 (24.5) | 66 (25.8) | 0.091 |
|  | Yes | 11 (57.9) | 179 (75.5) | 190 (74.2) |  |
|  | Total | 19 (100.0) | 237 (100.0) | 256 (100.0) |  |
|  |  |  |  |  |  |
| Clerical workers | No | 8 (66.7) | 22 (23.9) | 30 (28.8) | 0.002** |
|  | Yes | 4 (33.3) | 70 (76.1) | 74 (71.2) |  |
|  | Total | 12 (100.0) | 92 (100.0) | 104 (100.0) |  |
|  |  |  |  |  |  |
| Manual laborers | No | 9 (60.0) | 13 (26.0) | 22 (33.8) | 0.015* |
|  | Yes | 6 (40.0) | 37 (74.0) | 43 (66.2) |  |
|  | Total | 15 (100.0) | 50 (100.0) | 1. 100.0) |  |

Note: * P<0.05; **P<0.01. ^ PPE: personal protection equipment

Table S2. Association between availability of workplace policies and adequacy of workplace PPE storage by occupations

| Occupation | Availability of workplace policies for COVID-19 prevention | | | | |
| --- | --- | --- | --- | --- | --- |
|  |  | Not available | Available | Total | P value |
|  | Adequacy of workplace PPE^ storage | | | |  |
| Administrator and manager | No | 14 (70.0) | 72 (51.4) | 86 (53.8) | 0.119 |
|  | Yes | 6 (30.0) | 68 (48.6) | 74 (46.3) |  |
|  | Total | 20 (100.0) | 140 (100.0) | 160 (100.0) |  |
|  |  |  |  |  |  |
| Professionals | No | 23 (85.2) | 206 (67.5) | 229 (69.0) | 0.057 |
|  | Yes | 4 (14.8) | 99 (32.5) | 103 (31.0) |  |
|  | Total | 27 (100.0) | 305 (100.0) | 332 (100.0) |  |
|  |  |  |  |  |  |
| Associate professionals | No | 13 (81.3) | 135 (68.9) | 148 (69.8) | 0.300 |
|  | Yes | 3 (18.8) | 61 (31.1) | 64 (30.2) |  |
|  | Total | 16 (100.0) | 196 (100.0) | 212 (100.0) |  |
|  |  |  |  |  |  |
| Clerical workers | No | 7 (77.8) | 47 (62.7) | 54 (64.3) | 0.371 |
|  | Yes | 2 (22.2) | 28 (37.3) | 30 (35.7) |  |
|  | Total | 9 (100.0) | 75 (100.0) | 84 (100.0) |  |
|  |  |  |  |  |  |
| Manual laborers | No | 12 (92.3) | 19 (55.9) | 31 (66.0) | 0.018* |
|  | Yes | 1 (7.7) | 15 (44.1) | 16 (34.0) |  |
|  | Total | 13 (100.0) | 34 (100.0) | 47 (100.0) |  |

Note: * P<0.05; **P<0.01. ^ PPE: personal protection equipment

Table S3. Association between availability of workplace policies and information transparency on the infections occurred among co-workers and their family by occupations

| Occupation | Availability of workplace policies for COVID-19 prevention | | | | |
| --- | --- | --- | --- | --- | --- |
|  |  | Not available | Available | Total | P value |
|  | Information-sharing on the infections occurred among co-workers and their family | | | |  |
| Administrator and manager | No | 9 (50.0) | 29 (29.9) | 38 (33.0) | 0.096 |
|  | Yes | 9 (50.0) | 68 (70.1) | 77 (67.0) |  |
|  | Total | 18 (100.0) | 97 (100.0) | 115 (100.0) |  |
|  |  |  |  |  |  |
| Professionals | No | 14 (87.5) | 81 (35.1) | 95 (38.5) | <0.001** |
|  | Yes | 2 (12.5) | 150 (64.9) | 152 (61.5) |  |
|  | Total | 16 (100.0) | 231 (100.0) | 247 (100.0) |  |
|  |  |  |  |  |  |
| Associate professionals | No | 8 (72.7) | 58 (43.0) | 66 (45.2) | 0.056 |
|  | Yes | 3 (27.3) | 77 (57.0) | 80 (54.8) |  |
|  | Total | 11 (100.0) | 135 (100.0) | 146 (100.0) |  |
|  |  |  |  |  |  |
| Clerical workers | No | 4 (66.7) | 15 (32.6) | 19 (36.5) | 0.103 |
|  | Yes | 2 (33.3) | 31 (67.4) | 33 (63.5) |  |
|  | Total | 6 (100.0) | 46 (100.0) | 52 (100.0) |  |
|  |  |  |  |  |  |
| Manual laborers | No | 10 (83.3) | 8 (29.6) | 18 (46.2) | 0.002** |
|  | Yes | 2 (16.7) | 19 (70.4) | 21 (53.8) |  |
|  | Total | 12 (100.0) | 27 (100.0) | 39 (100.0) |  |

Note: * P<0.05; **P<0.01. ^ PPE: personal protection equipment
